# Antitumor activity of *Lycium barbarum* polysaccharides with different molecular weights: an *in vitro* and *in vivo* study

**DOI:** 10.1080/16546628.2017.1399770

**Published:** 2017-11-13

**Authors:** Xiangliang Deng, Xiangling Li, Shuang Luo, Yongyan Zheng, Xia Luo, Lian Zhou

**Affiliations:** ^a^ School of Chinese Materia Medica, Guangzhou University of Chinese Medicine, Guangzhou, PR China; ^b^ Research and Development Department, Infinitus Chinese Herbal Immunity Research Centre, Guangzhou, PR, China; ^c^ Research and Development Department, Guangdong Hybribio Co. Ltd, Guangzhou, PR, China

**Keywords:** *Lycium barbarum* polysaccharide, molecular weight, antitumor activity; H22 cells; flow cytometry; apoptosis

## Abstract

The antitumor activity of *Lycium barbarum* polysaccharide (LBP) has been reported, but the structure–bioactivity relationship has still not been fully elucidated. In this study, four water-soluble LBP fractions with serial different molecular weights (MWs) were separated from LBP, designated LBP-2, LBP-3, LBP-4, and LBP-5. After a characteristic analysis, the relationship between MW and antitumor activity of LBP was investigated both *in vitro* using murine hepatoma H22 cells and *in vivo* using H22 tumor-bearing mice. *In vitro*, the results showed that all the LBP fractions had significant inhibition on H22 cells, in which LBP-3 had the best activity. LBP-3 could induce apoptosis, mitochondrial membrane potential destruction, and S phase arrest in H22 cells. *In vivo*, the results showed that LBP-2, LBP-3, LBP-4, and LBP-5 could inhibit the tumor growth in H22 tumor-bearing mice by 18.18%, 37.97%, 9.09%, and 14.44%, respectively. However, only LBP-3 was able to decrease the tumor weight significantly in H22 tumor-bearing mice. Meanwhile, all the LBP fractions did not show significant toxicity to murine splenocytes, thymus, and spleen. Taken together, these results demonstrated that the antitumor activity of LBP was closely related to its MW, and LBP-3 with medium MW (40–350 kDa) was the main active fraction.

## Introduction

*Lycium barbarum* polysaccharide (LBP), which is extracted from the fruit of a well-known Chinese herb *Lycium barbarum*, is found to have antitumor activity in recent years. *In vitro* study results showed that LBP treatment inhibited tumor cells growth. Zhang [1] found that LBP treatment inhibited the growth of QGY7703 cells via induction of S-phase cell-cycle arrest and calcium-related apoptosis. Similar studies showed that LBP treatment could inhibit the growth of mouse colon cancer cells, human gastric cancer MGC-803, and SGC-7901 cells, and arrest the cell cycles at the G0/G1 or S phases [2,3]. The anticancer effects of LBP were also demonstrated in tumor-bearing mice. Gan [4] revealed that a polysaccharide–protein complex from *Lycium barbarum* could significantly inhibit the growth of transplantable sarcoma S180 and improved the immune system in S180-bearing mice. Another study led by Zhang [5] found that LBP could obviously depress the death rate of H22 tumor-bearing mice and diminish gross tumor volume and weight.

Although the anticancer effect of LBP has been demonstrated both *in vitro* and *in vivo*, various LBP samples, obtained by different methods, have different structures and molecular weight (MW) [6]. Recent studies have indicated that biological activities of the polysaccharide partly depend on its MW [7]. LBP has an MW range of 10–2300 kDa, and its antitumor activity is probably related to MW [7]. Zhang [8] observed the effects of LBP fractions on human liver cancer cells (SMMC-7721) and found that LBP-a4 with an MW of 10.2 kDa could inhibit the proliferation of SMMC-7721 cells in a dose- and time-dependent manner, but LBP-p8 with an MW of 6.50 × 10^3^ kDa could promote the growth of SMMC-7721 cells. However, the detailed relationship between the MWs and antitumor activities of LBP fractions is still not fully clear.

To further study the MW-antitumor activity relationship of LBP, LBP was separated into five fractions (LBP-1, LBP-2, LBP-3, LBP-4, and LBP-5) with serial different MWs by ultrafiltration membranes in this study. Then, the water-soluble LBP fractions (LBP-2, LBP-3, LBP-4, and LBP-5) were taken to investigate the antitumor activity both using murine hepatoma H22 cells *in vitro* and using H22 tumor-bearing mice *in vivo*.

## Materials and methods

### Materials

Penicillin–Streptomycin solution was purchased from Genom Biomedical Technology Co., Ltd (Hangzhou, China). Cisplatin Injection was purchased from NanJing Pharmaceutical Factory Co.,Ltd (Nanjing, China). Doxorubicin (Dox) for injection was purchased from Shenzhen Main Luck Pharmaceuticals Inc. (Shenzhen, China). Dimethyl sulfoxide (DMSO) was purchased from Tianjin Yongda Chemiacl Reagent Co., Ltd (Tianjin, China). Cyclophosphamide (Cy) for injection was purchased from Shanxi Powerdone Pharmaceutics Co., Ltd (Shanxi, China). RPMI-1640 was purchased from Corning; Rhodamine 123, propidium iodide (PI), 3-(4,5-dimethylthiazol-2-yl)-2,5-diphenyltetrazolium bromide (MTT), carbazole, galacturonic acid, and ribonuclease (RNase) were purchased from Sigma (St. Louis, USA). Annexin V-FITC/PI Apoptosis Detection Kit was purchased from eBioscience (Santa Clara, USA). Fetal bovine serum (FBS) was purchased from Biological Industries (Kibbutz Beit-Haemek, Israel).

### Preparation and characterization analysis of LBP fractions

LBP was prepared with hot water as described previously [9]. The LBP fractions with different series MW were prepared according to the relevant reference [8]. Briefly, the LBP fractions were isolated by ultrafiltration membranes with an MW cutoff (MWCO) of 3, 8, 40, 350, and 400 kDa successively. Based on the MW, the fractions were named LBP-1, LBP-2, LBP-3, LBP-4, and LBP-5, respectively. The powder of the fractions was obtained by freeze-drying. The water-soluble LBP fractions (LBP-2, LBP-3, LBP-4, and LBP-5) were taken for investigation in the following experiments.

The characterization of LBP fractions was analyzed as described previously [10–13]. Briefly, the total sugar was determined by the phenol-sulfuric acid method [10]. Protein content was measured by the Bradford method [11]. Uronic acid was measured by the sulfuric acid carbazole colorimetry method, and galacturonic acid was used as the standard [12]. The vibrations of molecules and polar bonds between the different atoms in LBP fractions were analyzed using IR (Avatar 30FT-IR infrared spectrometer, Nicnet) [13].

### Cell line and animals

Murine hepatoma H22 cells were cultured in RMPI-1640 with 10% FBS, 100 U/mL penicillin, and 100 μg/mL streptomycin at 37° with 5% CO_2_. The cells were subcultured until they reached the logarithmic growth phase.

Specific pathogen-free male BALB/c mice, weighing 20 ± 2 g, were purchased from the Guangdong Medical Laboratory Animal Center (Foshan, China). Animals were fed on a standard laboratory diet and water, and were kept in environmentally controlled quarters with temperature maintained at 25°C and a 12/12 h dark/light cycle. All experimental procedures were approved by the Animal Care and Use Committee of Guangzhou University of Chinese Medicine, PR China.

### In vitro *antitumor assay*

#### Cell viability assay

The effect of LBP fractions on cell viability was measured using the MTT assay. H22 cells in logarithmic growth phase were plated in 96-well plates (1 × 10^4^ cells/well) and treated with LBP-2, LBP-3, LBP-4, and LBP-5 (400, 800, 1200, and 1600 μg/mL) immediately for 24 h and 48 h. MTT (5 mg/mL) was added to each well at 24 h and 48 h respectively, and cells were incubated for another 4 h. After centrifugation at 300 × *g* for 10 min, the supernatant was removed, and 0.15 mL of DMSO was added to each well. The absorbance at 570 and 630 nm was measured on a microplate reader (Thermo Fisher Scientific, Waltham, USA). The percent viability of the treated cells was calculated using the following equation: relative viability (%) = (*A*_570 nm_ – *A*_630 nm_) sample / (*A*_570 nm_ – *A*_630 nm_) control × 100%.

In order to determine whether LBP fractions also have cytotoxicity to immune cells, the effects of LBP fractions on splenocytes viability were investigated in this study. Splenocytes were prepared as described previously with minor modifications [14]. Briefly, splenocytes were isolated from BALB/c mice, and the erythrocytes were lysed with red blood cell lysis buffer (0.829 mg/mL NH_4_Cl, 0.037 mg/mL Na_2_EDTA and 0.100 mg/mL KHCO_3_). After washing twice with precold phosphate-balanced solution (PBS), cells were resuspended in RMPI-1640 with 10% FBS, 100 U/mL penicillin, and 100 μg/mL streptomycin, and seeded in 96-well plates (4 × 10^5^ cells/well, sextuplicate wells in each group). After being treated with LBP fractions (400, 800, 1200, and 1600 μg/mL) for 72 h, the cell viability was measured by MTT assay as described above.

#### Cell-proliferation assay

The effect of LBP fractions on cell proliferation was detected by flow cytometry (FACSCanto II flow cytometer, BD Biosciences, Franklin Lakes, USA). H22 cells were treated with LBP-2, LBP-3, LBP-4, and LBP-5 (400, 800, 1200, and 1600 μg/mL) for 24, 48, and 72 h. Cells treated with RPMI-1640 medium were taken as negative controls, and those treated with cisplatin (10 μg/mL) were taken as positive controls. The cells were collected and washed twice with PBS before detection. Relative cell number was counted by flow cytometry for 1 min under a medium speed. The inhibition of LBP fractions on proliferation of H22 cells was calculated using the following equation: inhibition rate (%) = (cell number of negative control – cell number of sample) / cell number of negative control × 100%.

#### Apoptosis analysis

Cell apoptosis was analyzed using the Annexin V-FITC/PI apoptosis detection kit as described previously [15]. Briefly, after being treated with LBP-3 (400, 800, 1200 μg/mL) for 48 h, H22 cells were harvested and washed twice with PBS and stained with Annexin V-FITC/PI according to the manufacturer’s instructions. The samples were detected on an FACS Canto II flow cytometer (BD Biosciences) and analyzed using FACS Diva™ software.

#### MMP measurement

The MMP was detected by Rhodamine123 (Rho123), using flow cytometry and a fluorescence microscope (Nikon). After being treated with LBP-3 (400, 800, 1200 μg/mL) for 48 h, H22 cells were washed with PBS and dyed with Rho123 (10 μg/mL) at 37°C for 30 min. Morphological observations were carried out using a fluorescence microscope. The fluorescence intensity of cell was detected on an FACS Canto II flow cytometer (BD Biosciences) and analyzed with FACS Diva™ software.

#### Cell-cycle analysis

Cell cycles were analyzed by flow cytometry with PI staining as described previously [1]. Briefly, H22 cells were seeded in 24-well plates (5 × 10^4^ cells/well) and treated with LBP-3 (400, 800, 1200 μg/mL) for 72 h. The cells were harvested, washed twice with PBS, and fixed with 70% ethanol for 24 h at −20°C. After two washes with PBS, cells were stained with PI (10 μg/mL) and RNase (10 μg/mL) at room temperature for 30 min in the dark. The samples were detected by flow cytometry and analyzed with ModFit LT cell-cycle-analysis software.

### In vivo *antitumor assay*

#### Animal experiments and treatment protocol

To investigate the antitumor activity of LBP fractions *in vivo*, the murine H22 hepatocarcinoma model was used in this study. The animal experiments and treatment protocol were prepared as described previously with minor modifications [16]. Briefly, H22 cells (2 × 10^6^ cells/mouse) were injected into the right armpit subcutaneously in BALB/c mice. Twenty-four hours after the tumor inoculation of H22 cells, the mice were divided into the model group, LBP-2 group, LBP-3 group, LBP-4 group, and LBP-5 group. Mice were treated with LBP fractions intragastrically at a dose of 250 mg/kg, except for the model group. The treatments were administered once daily for 10 days. To further investigate whether LBP-3 could inhibit tumor growth in a dose-dependent manner, mice were divided into the model group, cyclophosphamide (Cy, 20 mg/kg) treatment group, Doxorubicin (Dox, 10 mg/kg) treatment group, and three doses of LBP-3 (62.5, 125 and 250 mg/kg) treatment group. Cy was given intragastrically once daily for 10 days. Dox was injected intraperitoneally on day 1. LBP-3 was given intragastrically as described above.

#### Tumor inhibition rate and immune organ index

Twenty-four hours after the last administration, mice were killed immediately by cervical dislocation after anesthesia with chloral hydrate. The tumor, spleen, and thymus were immediately dissected and weighed. The tumor inhibition rate and immune organ indexes were calculated using the following equation respectively: tumor inhibition rate (%) = (the average tumor weight of model group – the average tumor weight of LBP fraction group) / the average tumor weight of model group × 100%, spleen or thymus index (mg/g) = the weight of spleen or thymus (mg) / body weight (g).

### Statistical analysis

All values were expressed as mean ± SD. A one-way analysis of variance, followed by the Dunnett *t* test, was used to assess the statistical significance of differences between experimental groups. A *P* value of <0.05 was considered to be significant.

## Results

### Characterization of LBP fractions

The results showed that the total sugar contents of LBP-2, LBP-3, LBP-4, and LBP-5 were 66.88%, 54.47%, 56.53%, and 55.21%, respectively. The uronic acid, protein, and bound water were also found in the LBP fractions (Table 1).

**Table 1.** Components of LBP fractions.

Fractions	LBP-2	LBP-3	LBP-4	LBP-5
Molecular weights (kDa)	350–400	40–350	8–40	3–8
Total sugar (%)	66.88	54.7	56.53	55.21
Uronic acid (%)	11.03	8.11	10.65	10.14
Protein (%)	0.27	1.14	0.11	0.34
Bound water (%)	12.80	14.59	14.88	15.85
Fat (%)	4.30	11.10	7.25	2.10

The carbohydrate-related molecules, such as C–H and C＝C bonds, CH_3_ group, and glycosidic linkages, have characteristic absorptions in IR spectra [13]. Thus, IR spectra are usually used to identify the characteristic absorptions of polysaccharides. In the present study, the results showed that the IR spectra in all the LBP fractions appeared at about 3400, 2930, 1627, 1410, 1077, 918, 865, 818, and 778 cm^−1^ (Table 2). However, the absorption at 918 cm^−1^ was mainly found in LBP-4. The absorption peaks at about 3400 and 2930 cm^−1^ were due to hydroxyl stretching vibrations and C–H stretching vibrations, respectively [17,18]. The signal at about 1627 cm^−1^ was due to the bound water [17], consistent with the fact that the bound water was found in the LBP fractions. The absorption at 1410 and 1077 cm^−1^ was possibly due to nonsymmetric CH_3_ bending and C–O stretching vibration, respectively [13]. The small absorption band at 918 cm^−1^ was characteristic of β-glycosidic linkages between the sugars [18] and indicated that the β-glycosidic linkage mainly existed in the LBP-4. The characteristic absorption bands at 865 cm^−1^ and 818 cm^−1^ indicated that α-configuration and mannose existed in the LBP fractions [17,19]. The present results demonstrated that all the LBP fractions shared most of the characteristic absorptions.

**Table 2.** Results of IR analysis of LBP fractions.

Fractions	IR bands (cm^−1^)
LBP-2	3429, 2931, 1634, 1410, 1077, 865, 818, 778
LBP-3	3405, 2932, 1627, 1410, 1077, 866, 818, 778
LBP-4	3403, 2933, 1625, 1412, 1077, 918, 865, 818, 778
LBP-5	3411, 2932, 1625, 1412, 1077, 865, 818, 778

### LBP fractions were toxic to H22 cells, but not to murine splenocytes

**Figure 1.**
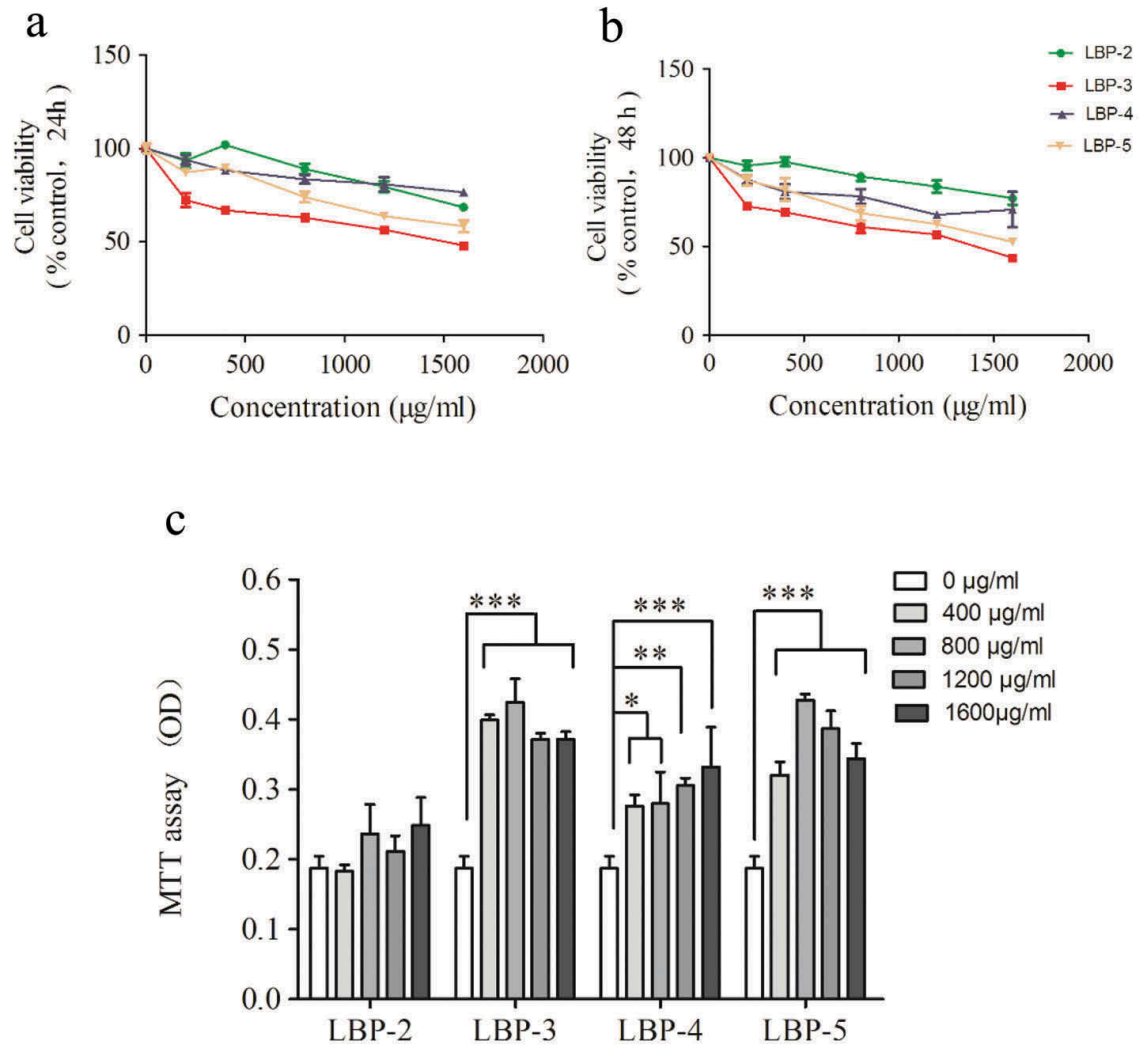
Effect of LBP fractions on the viability of H22 cells and splenocytes. H22 cells were treated with LBP fractions for 24 h (A) and 48 h (B). Splenocytes were treated with LBP fractions for 48 h (C). Cell viability was measured by MTT assay, and results are presented as percentage of control (untreated cells). Values are shown as the means ± SD of three replicates (*n *= 3) for H22 cells or six replicates (*n *= 6) for splenocytes. *P<0.05, **P<0.01 and ***P<0.001, compared with the control group (0 μg/mL).

To investigate the effect of the water-soluble LBP fractions (LBP-2, LBP-3, LBP-4, and LBP-5) on the viability of H22 cells, cells were measured by MTT assay after being treated with LBP fractions for 24 h and 48 h. The results showed that all the LBP fractions inhibited the viability of H22 cells in a dose-dependent manner (Figure 1(a,b)). Among these fractions, LBP-3 had the best inhibition with the lowest cell viability at the dose of 1600 μg/mL for 48 h.

To further confirm whether LBP fractions were also toxic to normal cells, the viability of murine splenocytes was measured by MTT assay after being treated with LBP fractions for 48 h. As shown in Figure 1(c), the results showed that LBP fractions were not toxic to murine splenocytes. On the contrary, LBP-3, LBP-4, and LBP-5 enhanced the cell viability. This was consistent with the previous studies that LBP could promote the proliferation of murine lymphocytes [20].

### LBP fractions inhibited the growth of H22 cells, and LBP-3 had the highest inhibition activity

**Figure 2.**
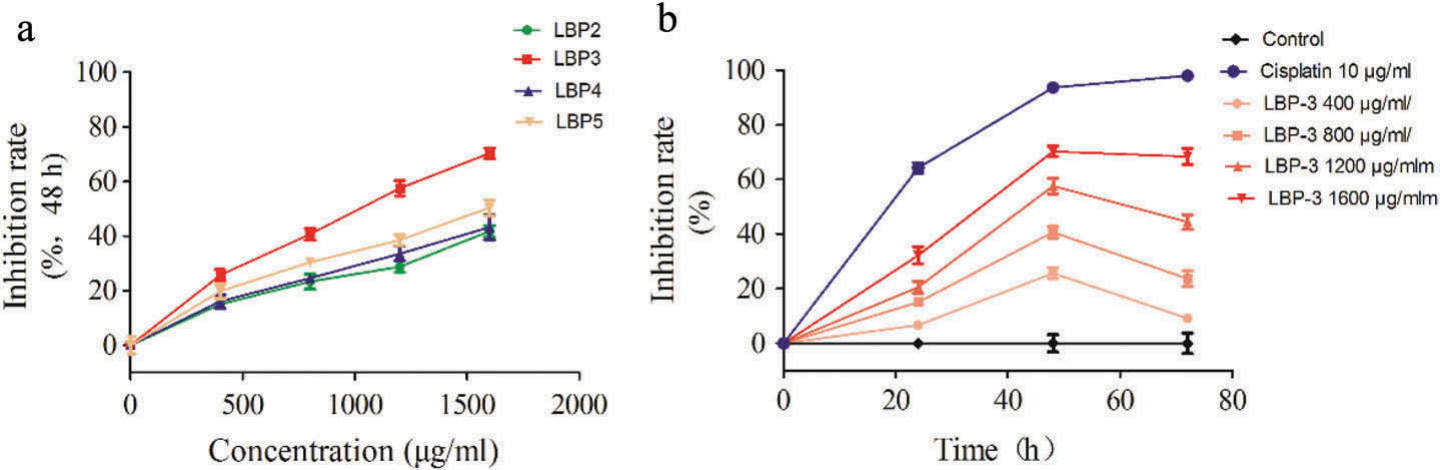
Effect of LBP fractions on H22 cells proliferation. (A) Cells were treated with LBP2-5 (400–1600 μg/mL) for 48 h. (B) Cells were treated with LBP-3 (400–1600 μg/mL) for 24–72 h. Cell numbers were counted by FCM, and results are presented as a percentage of control (untreated cells). Values are shown as the Means ± SD of three replicates (*n *= 3).

The effect of LBP fractions on H22 cells proliferation were measured by FCM. The results (Figure 2(a)) showed that all the LBP fractions could inhibit the growth of H22 cells in a dose-dependent manner. Among the four fractions, LBP-3 had the highest inhibition activity, especially at the dose of 1600 μg/mL for 48 h where the percentage of inhibition was over 60%. Further study showed that LBP-3 inhibited growth of H22 cells in dose- and time-dependent manners (Figure 2(b)).

Since LBP-3 had the highest inhibition activity on H22 cells, the following *in vitro* experiments were focused on LBP-3.

### LBP-3 induced apoptosis of H22 cells

**Figure 3.**
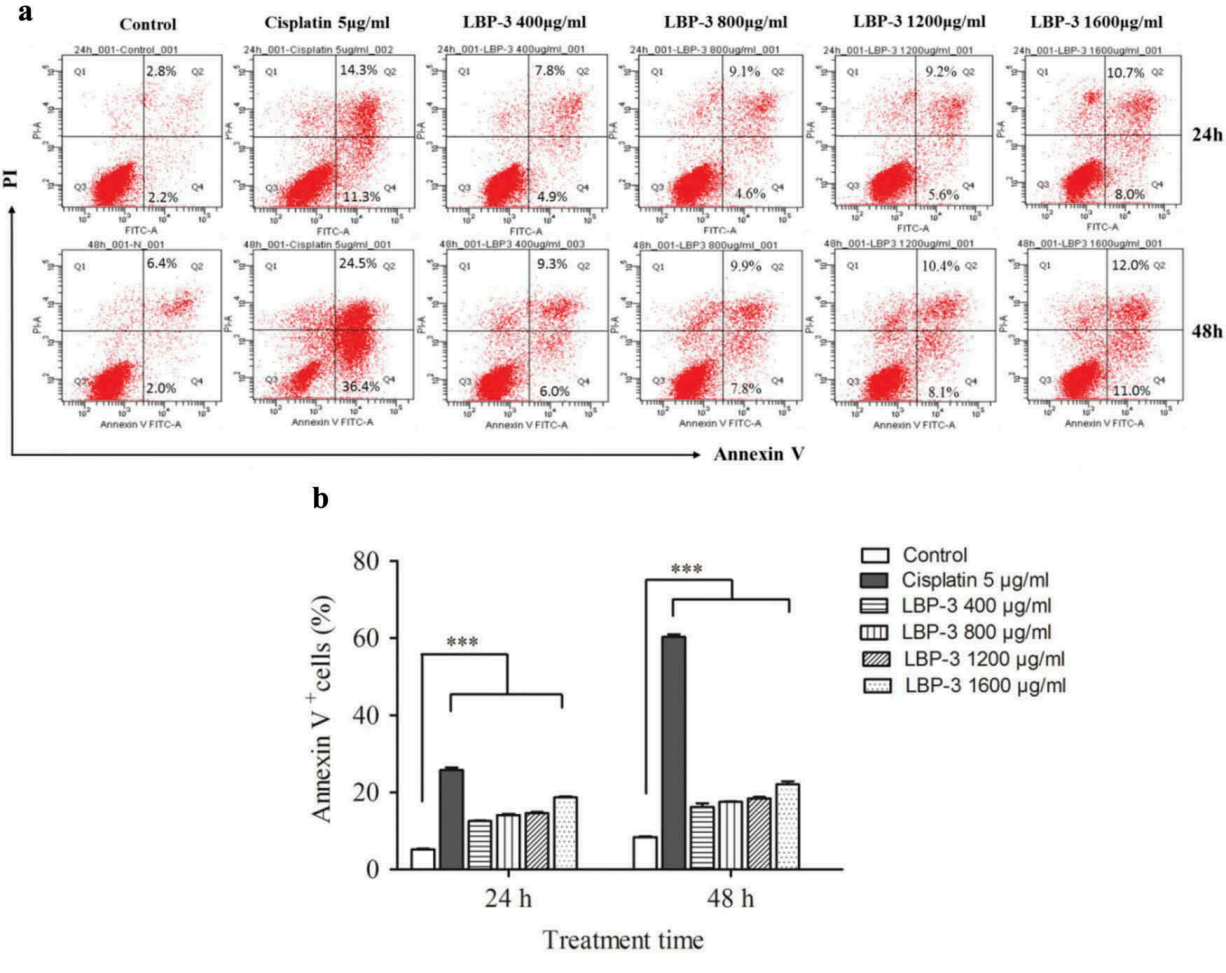
Effect of LBP-3 on H22 cells apoptosis. Cells were treated with LBP-3 (400–1600 μg/mL) for 24 and 48 h. Cells were stained by Annexin V/PI, and the apoptotic cell death (Annexin V+) was analyzed by FCM. Values are shown as the means ± SD of three replicates (*n *= 3). ***P<0.001, compared with the control group.

Annexin V-FITC/PI apoptosis detection kit is a standard method that is used to assess cell apoptosis [21]. A previous study showed that LBP could induce apoptosis of human hepatoma SMMC-7721 cells [6]. To determine whether LBP-3 could induce apoptosis of H22 cells, the apoptosis of cells was measured using the Annexin V-FITC/PI apoptosis detection kit using FCM after the cells were treated with LBP-3 for 24 h and 48 h (Figure 3). The results showed that LBP-3 significantly induced apoptosis of H22 cells in a dose-dependent manner.

### LBP-3 disrupted MMP on H22 cells

**Figure 4.**
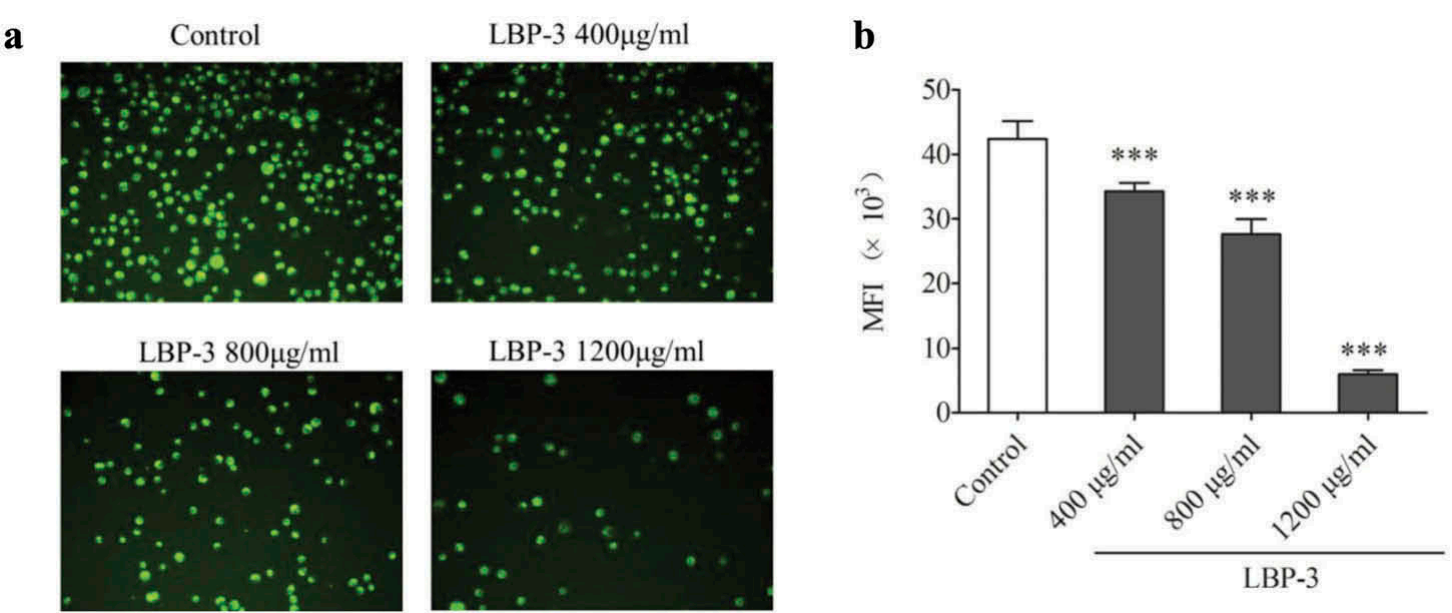
Effect of LBP-3 on H22 cells MMP. After being treated with LBP-3 (400–1200 μg/mL) for 48 h, cells were stained with Rho123 and observed in fluorescence microscopy with ×200 magnification (A). The Rho123 MFI of cells was measured by flow cytometry (B). Results of MMP are presented as mean of Rho123 MFI. Values were shown as the means ± SD of three replicates (*n *= 3). ***P<0.001, compared with the control group.

Since the mitochondrion is the powerhouse of the cell [22], disruption of the mitochondrial membrane potential (MMP) is one of the crucial factors in cell apoptosis [23] and has become a pharmacological target in cancer therapy for alterations in mitochondrial structure and functions that have long been observed in cancer cells [24]. Rhodamine-123 as a cationic fluorescent probe has been used for the measurement of MMP [25]. To investigate whether LBP-3-induced apoptosis of H22 cells was related to the MMP disruption, H22 cells were treated with different concentrations of LBP-3 for 48 h. Then, the MMP was measured using Rho123 as a fluorescent probe with fluorescence microscopy and FCM, respectively. The fluorescence microscopy results showed that Rho123 accumulation in H22 cells decreased significantly after treatment with LBP-3 (Figure 4(a)). The FCM analysis results showed that LBP-3 disrupted the MMP of H22 cells in a dose-dependent manner (Figure 4(b)).

**Figure 5.**
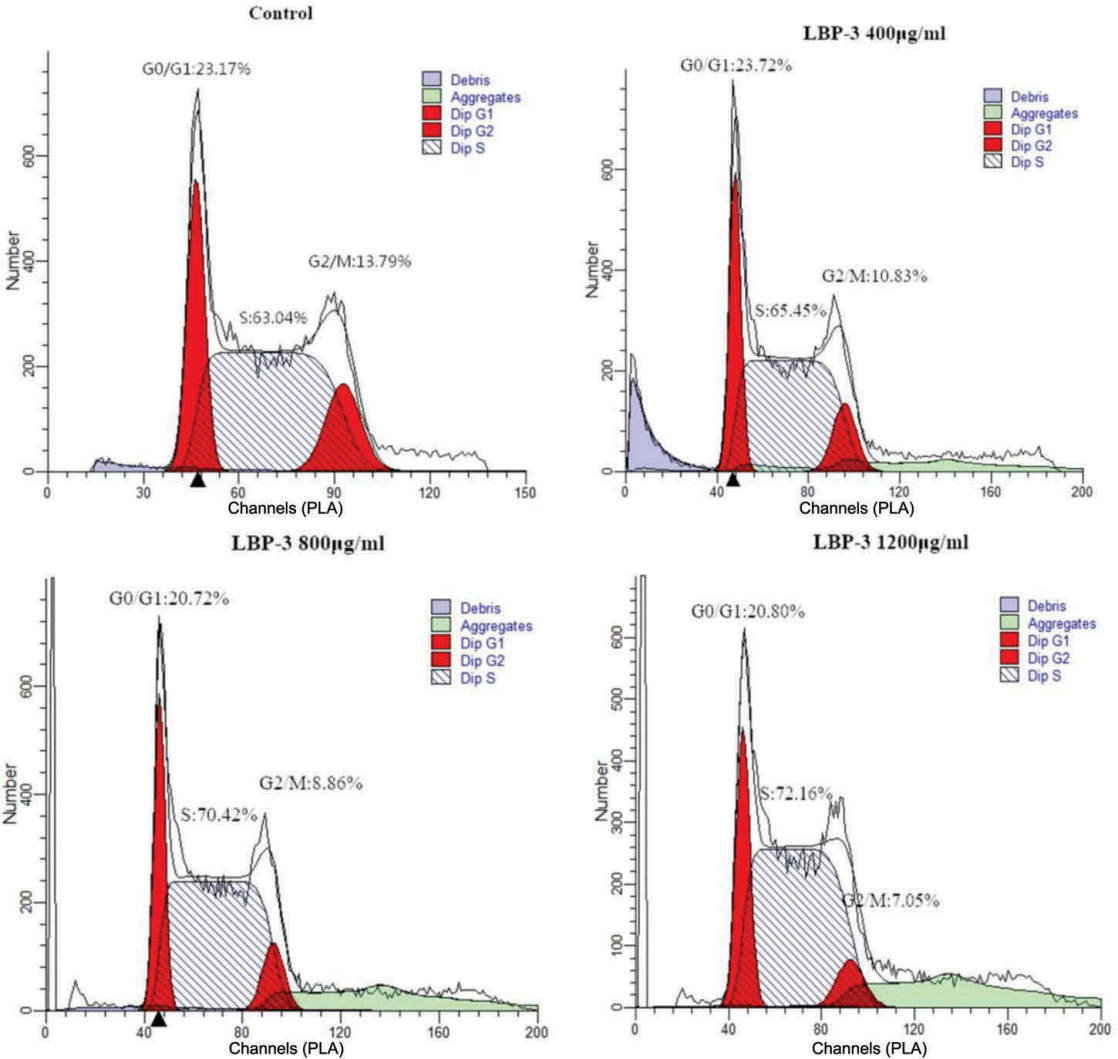
Inhibition of cell-cycle progress in H22 cells by treatment with LBP-3. H22 cells were treated with LBP-3 or medium for 72 h. Cells were fixed with 70% ethanol and stained with PI, and then the cell-cycle distribution was analyzed by flow cytometry.

### LBP-3 arrested H22 cells at the S phase

Previous studies had shown that LBP could arrest different human cancer cell lines at different phases [1–3]. To investigate whether LBP-3 inhibited H22 cell proliferation was related to arrest of cell-cycle progression, cell-cycle distribution of H22 cells was assayed by FCM after being treated with LBP-3 (400, 800, and 1200 μg/mL) for 72 h. The results showed that LBP-3 treatment caused a significant increase in cell number in the S phase (Table 3 and Figure 5), suggesting that LBP-3 could arrest H22 cells at the S phase directly.

**Table 3.** Effect of LBP-3 on cell-cycle progression in H22 cells.

Groups	Concentration (μg/mL)	G0/G1 phase (%)	S phase (%)	G2/M phase (%)
Control	0	23.83 ± 0.58	63.7 ± 0.96	12.48 ± 1.32
LBP-3	400	23.82 ± 0.09	65.86 ± 1.07*	10.59 ± 0.67
	800	21.49 ± 0.46***	70.41 ± 0.72***	8.03 ± 0.82***
	1200	21.17 ± 0.37***	72.25 ± 0.1***	6.58 ± 0.47***

Data are shown as mean ± SD (*n* = 3). *P < 0.05, **P < 0.01, ***P < 0.001 vs the control group.

### Effects of LBP fractions on tumor growth, thymus, and spleen index in H22 tumor-bearing mice

In order to confirm the results that LBP-3 had the highest antitumor activity as shown above among the LBP fractions, the effects of LBP fractions on tumor growth in H22 tumor-bearing mice were investigated. Since tumor-mass reduction often indicates a strong antitumor effect [16], tumor inhibition rate as an indicator was used to evaluate the antitumor activity of LBP fractions in the present study. The results showed that LBP-2, LBP-3, LBP-4, and LBP-5 could inhibit tumor growth in H22 tumor-bearing mice by 18.18%, 37.97%, 9.09%, and 14.44%, respectively. However, only LBP-3 significantly decreased the tumor weight in the mice (Table 4). In the further study, we found that LBP-3 inhibited the tumor growth in a dose-dependent manner (Table 5).

**Table 4.** Effect of LBP fractions on tumor growth, thymus index, and spleen index in H22 tumor-bearing mice.

Groups	Dose (mg/kg)	Tumor weight (g)	Inhibition rate (%)	Spleen index (mg/g)	Thymus index (mg/g)
Model	0	1.87 ± 0.65	–	7.08 ± 1.05	1.32 ± 0.39
LBP-2	250	1.53 ± 0.37	18.18	6.66 ± 1.59	1.35 ± 0.33
LBP-3	250	1.16 ± 0.40*	37.97	7.25 ± 1.81	1.44 ± 0.29
LBP-4	250	1.70 ± 0.43	9.09	7.99 ± 0.43	1.52 ± 0.34
LBP-5	250	1.60 ± 0.72	14.44	7.33 ± 1.17	1.41 ± 0.34

Data are shown as mean ± SD (*n* = 10). *P < 0.05 vs the model group.

**Table 5.** Effect of LBP-3 on tumor growth and thymus index in H22 tumor-bearing mice.

Groups	Dose (mg/kg)	Tumor weight (g)	Inhibition rate (%)	Thymus index (mg/g)
Model		1.63 ± 0.54	–	1.05 ± 0.27
Cy	20	0.86 ± 0.25**	47.24	0.38 ± 0.11***
Dox	10	0.24 ± 0.09***	85.28	0.15 ± 0.06***
LBP-3	62.5	1.23 ± 0.35	24.54	1.04 ± 0.19
	125	1.15 ± 0.51	29.45	1.16 ± 0.21
	250	0.90 ± 0.40**	44.79	1.13 ± 0.30

Data are shown as mean ± SD (*n* = 8). **P < 0.01, ***P < 0.001 vs the control group.

Accumulating evidence had demonstrated that chemotherapy drugs often resulted in immunosuppressive side-effects [26–30]. To investigate whether LBP fractions could cause immunosuppressive side-effects in H22 tumor-bearing mice, the effects of LBP fractions on the spleen and thymus index were investigated in the present study. As expected, Dox and Cy significantly reduced the thymus index of the mice (Table 5). However, the LBP fractions had no significant effect on the spleen and thymus index of the mice (Tables 4 and 5). The results indicated that the LBP fraction did not cause immunosuppressive side-effects in the H22 tumor-bearing mice.

## Discussion

In the present study, four water-soluble LBP fractions with serial different MW were isolated from LBP, in which we found that LBP-3 with medium MW had the highest antitumor activity in both *in vitro* and *in vivo* studies.

Biological activities of the polysaccharide partly depend on its MW [7]. Polysacchrides from *Lycium barbarum* have been demonstrated to have anticancer activity both *in vitro* and *in vivo*. However, LBP has an MW range of 10–2300 kDa [7], and the detailed relationship between the MW and antitumor activities of LBP fractions is still unclear. To clarify this, LBP were cut into five fractions with a series of different MWs, and the four water-soluble fractions were taken for investigation in the present study. Firstly, we investigated the effects of LBP fractions on viability and proliferation of H22 cells *in vitro*. Although all the LBP fractions could inhibit the viability and proliferation of H22 cells, LBP-3 had shown the best activity. Secondly, we investigated the effect of LBP fractions on tumor growth in H22 tumor-bearing mice to confirm the *in vitro* results. In accordance with the study *in vitro*, LBP3 had the best antitumor activity among the four LBP fractions *in vivo*. To our knowledge, this is the first investigation of antitumor activity in an LBP fraction with a series of different MWs both *in vitro* and *in vivo* at the same time. The results indicate that LBP-3 is the main active fraction of LBP in antitumor activity.

Apoptosis and cell-cycle arrest are both mechanisms for cytotoxic anticancer drugs work in cancer treatment [31,32]. Previous studies had shown that LBP could induce cancer cell apoptosis and cell-cycle arrest [1–5]. In our study, we found that LBP3 disrupted MMP and induced H22 cell apoptosis. The mitochondrion is the powerhouse of the cell [22], and we conclude that LBP-3 induced H22 cell apoptosis partly by disrupting MMP. Furthermore, LBP-3 could cause H22 cell arrest at the S phase. This agrees with several previous reports that LBP caused S phase arrest in human hepatoma QGY7703 cells [1] and gastric cancer SGC-7901 cells [3]. However, the other studies showed that LBP caused G0/G1 phase arrest in gastric cancer MGC-803 cells [3] and colon cancer cells [2]. These indicate that multiple mechanisms may be responsible for the anticancer effects of LBP in different types of cancer cells [2].

It is known that cytotoxic chemotherapy drugs not only kill cancer cells but also kill normal cells, which causes toxic side effects [33]. Myelosuppression and immunosuppression are common side effects for most chemotherapy drugs. Since the immune system plays an important role in preventing pathogen invasion, immunosuppression often leads to the development of opportunist infections, and even significant morbidity and mortality [30]. In our study, we found that none of the LBP fractions were cytotoxic to splenocytes, and furthermore LBP3, LBP4, and LBP5 could improve cell viability. In accordance with the *in vitro* study, none of the LBP fractions suppressed the thymus or spleen index in H22-tumor-bearing mice. In fact, LBP is extracted from the edible Chinese herb, *Lycium barbarum*. Previous studies have shown that LBP could enhance the proliferation of splenocytes in mice [34,35], activate macrophages [36], and promote both the phenotypic and functional maturation of DC [37]. Besides, LBP also has various other biological activities, such as neuroprotective effects [38,39], radioprotective activity [40], cardioprotective activity [41], and hepatoprotective effects [42,43]. Clinical trials have shown that LBP had remarkable protective effects in patients with type 2 diabetes [44]. These indicate that LBP-3 might be safe for cancer treatment. In order to support our conclusion, we further investigated the effect of LBP-3 on liver and kidney in H22-tumor-bearing mice. Results showed that LBP-3 did not cause damage in either liver or kidney tissue (**Figure S1 in the supplemental file**). Meanwhile, we found that LBP-3 did not cause the serum levels of AST, ALT or BUN increase (**Table S1 in the supplemental file**) which often indicates the dysfunction of liver and kidney, respectively. Taken together, we conclude that LBP-3 may be safe for antitumor therapy.

## Conclusions

In the present study, the MW–antitumor activity relationship of LBP was investigated both *in vitro* and *in vivo*. In summary, four water-soluble LBP fractions (LBP-2, LBP-3, LBP-4, and LBP-5) with serial different MWs were separated from LBP by ultrafiltration membranes. *In vitro*, LBP-3 had the highest inhibition activity on H22 cells among the four fractions. LBP-3 induced apoptosis, MMP destruction, and S phase arrest in H22 cells, but it did not inhibit the cell viability of murine splenocytes. Consistent with the results *in vitro*, LBP-3 had the highest activity in inhibiting tumor growth and did not suppress the thymus and spleen index in H22-tumor-bearing mice. These results demonstrated that the antitumor activity of LBP was closely related to its MW. LBP-3 with medium MW was the main active fraction of LBP in antitumor therapy, and it seemed to be safe for use in antitumor therapy.

## Supplementary Material

Supplementary_data.docx
