# Supplementary material for: Antitumor activity of Lycium barbarum polysaccharides with different molecular weights: an in vitro and in vivo study
Source: Food Nutr Res. 2017 Nov 13;61(1):1399770. doi: 10.1080/16546628.2017.1399770 (PMC6516794; doi:10.1080/16546628.2017.1399770)
Supplement: Supplementary_data.docx [file ZFNR_A_1399770_SM3670.docx]

Supplementary data (Figure s1 and Table s1) for Antitumor activity of *Lycium barbarum* polysaccharides with different molecular weight: an *in vitro* and *in vivo* study


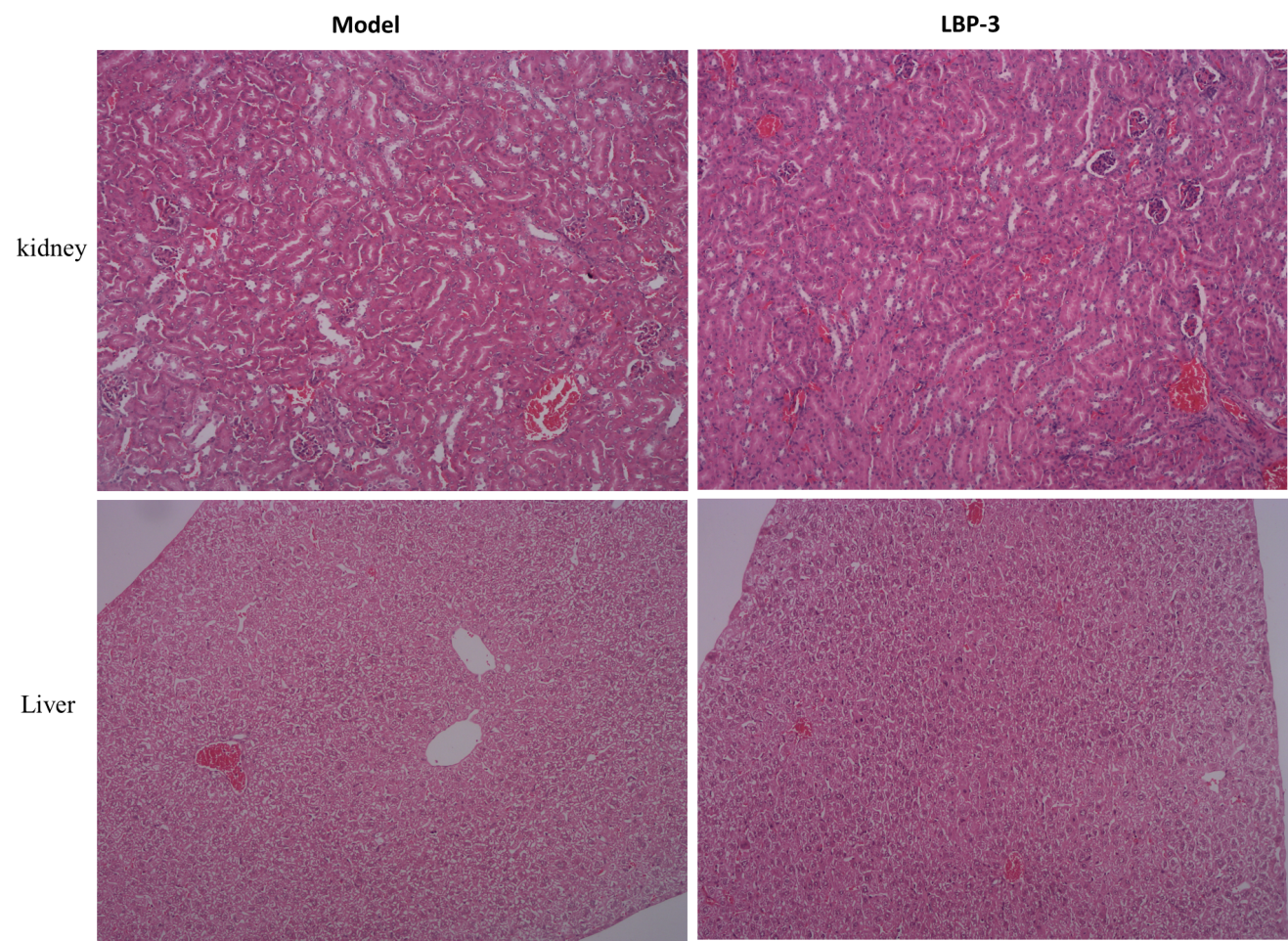


**Figure s1**.Representative photomicrographs of kidney and liver tissue from H22 tumor-bearing mice. The mice were treated with LBP-3 (250 mg/kg) in LBP-3 group. Hematoxylin and eosin (H&E) stain (magnification 100×).

**Table s1**

Effect of LBP-3 on the serum levels of AST, ALT and BUN in H22 tumor-bearing mice

| Groups | AST (U/L) | ALT (U/L) | BUN (mmol/L) |
| --- | --- | --- | --- |
| Model | 12.94±8.77 | 6.20±1.94 | 8.70±0.83 |
| LBP-3 | 13.30±3.13 | 6.84±3.57 | 6.88±1.10** |

Notes: Data were shown as Mean ± SD (*n* = 8). ^**^ *P* < 0.01 *vs* the control group. AST, aspartate aminotransferase; ALT, alanine aminotransferase; BUN, blood urea nitrogen.
